# Strength of socio-political attitudes moderates electrophysiological responses to perceptual anomalies

**DOI:** 10.1371/journal.pone.0220732

**Published:** 2019-08-05

**Authors:** Stefan Reiss, Johannes Klackl, Travis Proulx, Eva Jonas

**Affiliations:** 1 Dept. of Psychology, University of Salzburg, Salzburg, Austria; 2 School of Psychology, Cardiff University, Cardiff, United Kingdom; Universidad Complutense Madrid, SPAIN

## Abstract

People with strong (vs. moderate) political attitudes have been shown to exhibit less phasic reactivity to perceptual anomalies, presumably to prevent their committed meaning systems from being challenged by novel experiences. Several researchers have proposed that (but not tested whether) firmly committed individuals also engage in more attentional suppression of anomalies, likely mediated by prestimulus alpha power. We expected participants with strong (vs. moderate) political attitudes to display increased pre-stimulus alpha power when processing perceptual anomalies. We recorded electrophysiological activity during the presentation of normal cards (control group) or both normal and anomalous playing cards (experimental group; *total N* = 191). In line with our predictions, the presence of anomalous playing cards in the stimulus set increased prestimulus alpha power only among individuals with strong but not moderate political attitudes. As potential markers of phasic reactivity, we also analyzed the late positive potential (LPP) and earlier components of the event-related potential, namely P1, N1, and P300. The moderating effect of extreme attitudes on ERP amplitudes remained inconclusive. Altogether, our findings support the idea that ideological conviction is related to increased tonic responses to perceptual anomalies.

## Introduction

Recently, a branch of psychological research has emerged that deals with biological correlates of political attitudes and orientation. *Political neuroscience* combines political science, psychology and cognitive neuroscience [1] to investigate whether biological or even genetic factors underlie interindividual differences in political orientation [2]. Differences between liberals and conservatives have been reported in a variety of experiments, a key factor therein being *negativity bias* [3]. For instance, liberals have been hypothesized as being more sensitive to cognitive conflict than conservatives, as indicated by higher error-related negativity (ERN) amplitude in a Go/No-Go task [4]. In contrast, conservatives have been found to react more strongly to threatening and emotionally arousing stimuli [5,6].

### The role of strong political views in perception and processing of anomalies

There is research that suggests that it is not the content of worldviews and belief systems that defines individuals’ reactions to anomalies, inconsistencies, expectancy violations and committed errors, but rather the rigidity and conviction with which they cling to these frameworks. Research in the 1960s started investigating the similarities of conservatives and liberals, arguing that *extremists* on both sides of the political spectrum tend to make more stereotypical attributions and find earlier perceptual closure than politically neutral individuals [7]. As such, Brandt et al. [8] stated that ‘both liberals and conservatives will dig in their heels to defend their ideological values and beliefs’ when experiencing threatening stimuli (p. 307). This statement seems to be valid beyond social attitudes, as illustrated by findings on religiosity. Inzlicht and colleagues found differences in ERN after errors in a Stroop test were attenuated in those high (vs. moderate) in religious commitment. These individuals appeared to shift attention away from potentially anxiety-evoking stimuli, presumably to reduce uncertainty and error [9,10].

Individuals with strong attitudes—either liberal or conservative—have fortified belief systems that subjectively need to be protected from attacks. The idea that relative attitude strength (the deviation from neutrality) is more important than the attitude content is supported by the finding that participants tend to affirm their pre-existing attitudes–be they liberal or conservative–when confronted with threats such as mortality salience [11,12], challenges to their worldviews [13] or meaning violations [14]. This view of political and attitude rigidity implies higher rigidity on either end of the political spectrum than in centrist beliefs. As radical political stances require “drawing political frontiers and defining an adversary” [15], centrist and moderate beliefs are by nature less rigid than those on the far ends of the spectrum.

### Meaning maintenance and general threat model

More generally, humans construe frameworks and schemas to predict experiences. The meaning maintenance model or *MMM* [16–18] defines *meaning* as expected relationships between people, places, objects and ideas construed to make the world predictable. Experiences that are at odds with these frameworks lead to feelings of uncertainty, *disanxiousuncertlibrium* [18,19], entropy [20] or anxiety [20]. These effects have been found even for subtle, non-conscious expectancy violations like having people read passages of absurdist literature, humor and art [21–23], or covertly swapping experimenters without participants noticing [24].

Another subtle violation of expectancies is the presentation of reverse-colored playing cards [25]: when asked to indicate the parity or suit of playing cards with anomalous colors such as a black queen of hearts or red three of clubs, some participants showed increased signs of distress and arousal. Most participants first did not explicitly notice the manipulated cards but showed ‘perceptual denial’ [24], suggesting that they assimilated the cards to match existing schemas. Despite being a task-irrelevant feature and remaining undetected by participants, anomalous cards increased pupil dilation (PD) compared with a control condition where only normal cards were presented, indicating some form of physiological response [26]. Furthermore, Sleegers et al. [26] showed that participants with strong political attitudes—either liberal or conservative—exhibited smaller pupillary dilation responses to the presentation of anomalous playing cards.

The findings seem to converge on one possible explanation: that individuals with strong ideological convictions, regardless of ideological orientation, allocate less attention to anomalies, arguably by means of attentional suppression [9,27]. However, this hypothesis has yet to be directly investigated. Thus, in this paper, we aim to gauge the influence of attitude strength on an electroencephalography (EEG) measure that is closely related to attentional suppression: spectral power in the alpha frequency band (8–13 Hz).

### Two ways of addressing expectancy violations

When encountering expectancy violations, one may distinguish between at least two basic kinds of neuronal processes, namely phasic and tonic processes. Phasic neural processes reflect post-stimulus reactivity to the “odd” attributes of the perceived stimuli and trigger discrepancy-related neural markers such as activation of the anterior cingulate cortex (ACC), which is commonly related to error detection, higher ERN amplitude, and increased pupil dilation [9,26,28]. On the other hand, inconsistencies may also instigate tonic processes such as suppression, which enable assimilation, that is, (mis-)perceiving the inconsistent stimulus as being consistent with a pre-existing framework.

With expectancy violations that typically go unnoticed, such as anomalous playing cards, tonic neuronal processes may even prevent phasic reactivity. As a result, inconsistent stimulus features (i.e. suit/color mismatch) may not become salient to participants. In this example, one may argue that expectations about correspondences between colors and suits of playing cards (hearts are red and spades are black) remain unaltered, despite observing cards that do not match these expectations. In other terms, phasic processes that serve to detect inconsistencies may often fail to appear due to tonic attentional suppression.

According to our view, the dominance of tonic inhibitory over phasic reactive processes should be especially prevalent among individuals with stronger, more rigid meaning frameworks (i.e. political beliefs). For this research, the most important EEG measure is prestimulus alpha power which represents tonic attentional suppression. We also investigate ERP components for exploratory reasons.

#### Prestimulus alpha power and attentional suppression

The alpha frequency band (8–13 Hz) is heavily involved in the facilitation and inhibition of stimulus processing [29–33]. Cortical oscillations in this frequency band are inversely correlated with how much attention individuals devote to stimuli, and how much neural activation these stimuli produce. Thus, higher alpha power is related to greater inhibition of stimulus processing [34–36]. For example, lower prestimulus alpha power predicts better performance in identifying briefly presented letters [29,30]. In contrast, higher prestimulus alpha power over parieto-occipital regions has been associated with *worse* performance in a visual contrast discrimination task; this has been interpreted as reflecting inhibition of visual processing [33].

When anticipating sensory input, alpha power in task-relevant cortical regions decreases, presumably to release inhibition and facilitate information intake. For example, alpha power has been shown to decrease when the appearance of a target stimulus can be anticipated; alpha power was lowest right before the anticipated stimulus onset, suggesting top-down biasing in cortical excitability through disinhibition [32]. Conversely, blocking out irrelevant information reflects an increased prestimulus alpha power: when expecting auditory stimuli, alpha power in (task-irrelevant) occipital regions increases, indicating functional inhibition [37]. In the same vein, alpha power is increased in cortical areas corresponding to parts of the visual field in which visual input needs to be ignored [31]. To summarize, prestimulus alpha seems to be an indicator of how much attention is deployed prior to stimulus onset [38,39].

#### Event-related potentials (ERP) and reactive processing

Despite our main interest being spectral alpha power, we are also interested in instantaneous, phasic responses to expectancy violations. We will thus investigate event-related potentials in exploratory analyses. Previous research has shown differences in pupillary reactions to anomalous playing cards depending on political extremism [26]. However, the relation between pupil dilation (PD) and ERP measures is not straight forward: experiments investigating paradigms such as auditory [40] or visual oddball tasks [41] reported no correlation between PD and the P300 component, suggesting that ERP components and PD are not functionally equivalent in indicating the underlying phasic processes.

The late positive potential (LPP) is an ERP component that is hypothesized to reflect attention to motivationally significant stimuli. It is most prominent along centroparietal sites and is larger for both pleasant and unpleasant stimuli compared to neutral stimuli [42]. In threat-and-defense research, LPP has been shown to be higher for threat-related words than unpleasant but threat-unrelated words, pleasant words, and neutral words [43,44]. The LPP also has been proposed to indicate the affective significance of a stimulus through motivational activation of appetitive and defensive systems [45]. Thus, the LPP seems to reflect both top-down and stimulus-driven bottom-up processes–the presence of motivationally relevant stimuli increases the allocation of attention to them. However, the correlation between LPP and PD has been inconclusive for both novel and emotional stimuli [46].

Given the findings of Sleegers et al. [26] that politically extreme individuals react to perceptual anomalies with attenuated pupillary responses, and the assumptions of attentional gating, our hypothesis in this study is as follows: Participants with strong political stances (compared with participants with a moderate stance) will show *increased* prestimulus alpha power when presented with anomalous cards.

To summarize, this paper aims to explore electrocortical correlates of perceptual anomalies and how they are moderated by political attitudes and values. We chose an ethnocentrism measure for the attitude assessment since at the time of recording the data, the refugee situation was topical in Europe [47,48]. Some individuals regarded the emigration of refugees from the Middle East as a potential threat to their national or ethnic group, whereas others supported immigration to Europe because they saw it as a moral imperative and clung less to the idea of having to defend their own ethnic group. The existence of this controversy seemed to make ethnocentrism an ideal value upon which to calculate the strength of socio-political attitudes. Bizumic and colleagues defined ethnocentrism as ‘a sense of ethnic self-centeredness and self-importance’ that involves seeing one's own group as more important that other groups, and more important than its individual members [48].

We expected participants with strong (vs. moderate) attitudes to show higher prestimulus alpha power when confronted with expectancy-violating stimuli. Furthermore, we analyzed the ERP components P1, N1, P300, and LPP to investigate potential indices of increased phasic neural responses to the same expectancy-violating stimuli.

## Method

Ethical approval for the research was granted by the ethics committee of the Paris Lodron University Salzburg and participants gave their written informed consent to participate in the study. All data and the analysis script can be found in S1 Dataset.

### Participants

Data were collected in two waves across two academic years. The final sample consisted of 191 participants (115 female, 22 undisclosed; aged 22.99 ± 3.60 years): 98 participants were presented with only normal cards (control group) and 93 participants were presented with both normal and anomalous cards (experimental group). All participants gave informed consent and could interrupt their participation in the study at any point; no participant withdrew from the experiment.

### Materials and procedure

#### Scales and measures

Participants were seated in a comfortable chair in the laboratory, and EEG electrodes were applied. All questionnaires and tasks were presented using Inquisit 4.0.8 [49] on a 15.6-inch screen with 1980 × 1080 pixel resolution. After the briefing, a 3-min baseline EEG recording (to control for interindividual differences in alpha power) and some filler tasks, participants completed the card task, described in more detail below. Following the card task, participants filled out an ethnocentrism questionnaire. Handedness and demographic data were assessed. Participants were debriefed and rewarded with money or partial course credit.

**Fig 1:**
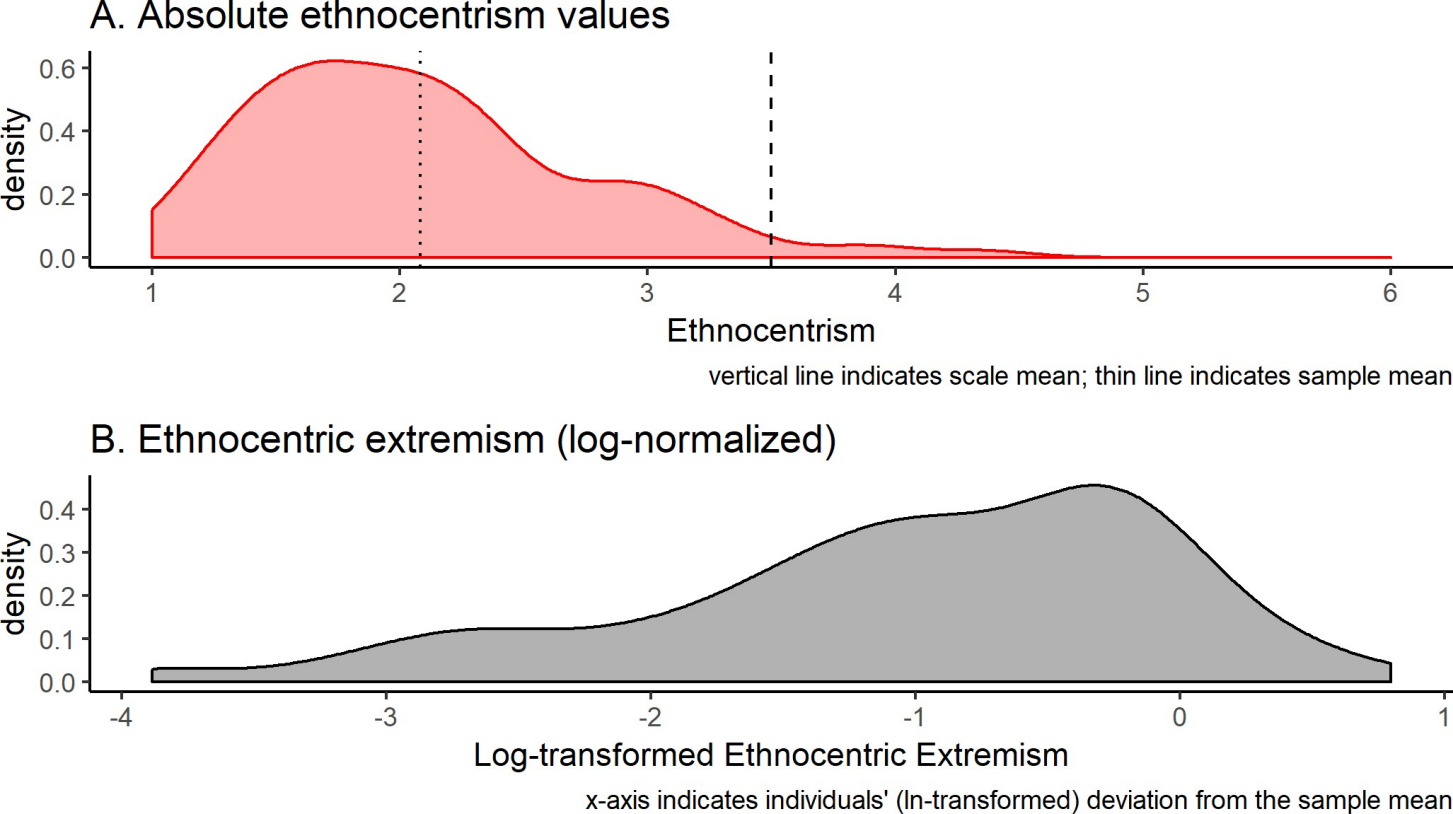
Distribution of ethnocentrism scores. (A) Absolute ethnocentrism values and (B) log-normalized deviations from sample mean.

Ethnocentrism. To gauge participants’ political attitudes, we used a ten-item scale previously employed by Agroskin and Jonas [50]: the scale consists of six items from the 60-item ethnocentrism scale by Bizumic et al. [51], and four items reflecting anti-immigrant attitudes [50]. Exemplary items were “No matter what, I will always support my cultural or ethnic group and never let it down” and “My country’s economy should be protected against mass immigration”. All items were rated on a six-point likert scale (α = .75, *M* = 2.08, *SD* = 0.67). Lower values indicate attitudes more socially liberal than conservative. The sample mean was significantly lower than the scale midpoint; *t*(190) = -29.26, *p* < .001. We calculated strength of political attitude as the difference in value of each participant’s ethnocentrism score from the sample mean; thus, a lower value indicates moderate attitude strength, and a high value indicates a large deviation from the sample mean, either toward low or high ethnocentrism. As the deviation measure was not normally distributed, we log-transformed it to approach normal distribution (see Fig 1).

**Fig 2:**
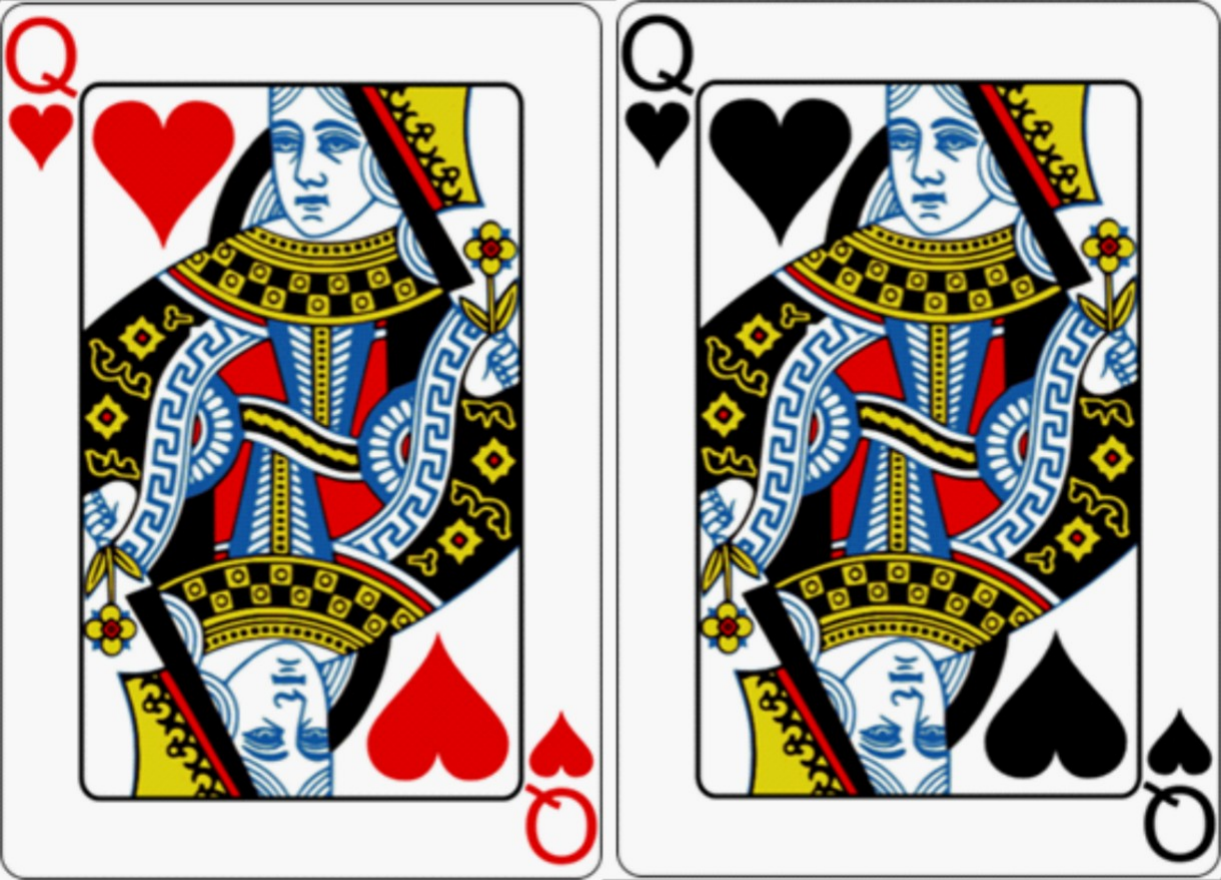
Stimuli of the card task. Examples for normal (left) and anomalous playing cards (right) used in the experiment.

**Fig 3:**
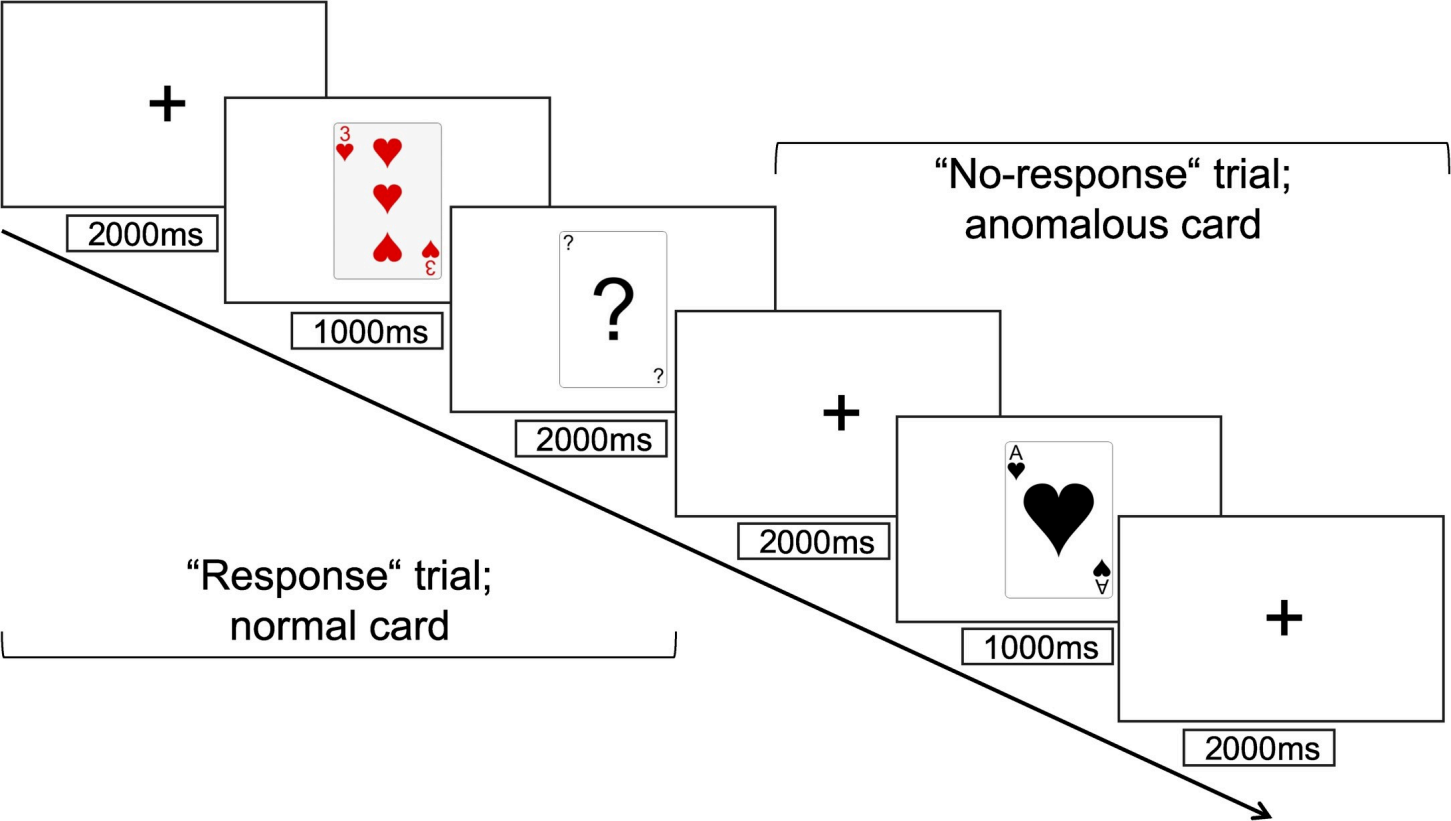
Time course of trials with and without response.

Card task. We used a mixed design for our experiment: Participants were assigned to the experimental or the control group in a between-subjects fashion. For participants in the control group, all trials consisted of regularly colored playing cards. In the experimental group, half of the stimuli were reverse-colored, that is, anomalous playing cards, and t half were normal playing cards. This created the opportunity for a within-subjects comparison within the experimental group (Fig 2). To test our hypotheses, we focused on the between-subjects manipulation. For exploratory purposes, we will also report the effects for the within-subjects manipulation. Each participant was presented with 180 trials. For the second wave of participants (*n*_2_ = 112), we adapted the procedure to accommodate another study not relevant to this report. Therefore, we reduced the trial count in the card task to 140 trials (56 probe trials). A schematic representation of the trials can be seen in Fig 3. In each trial, a fixation cross was present in the center of the screen for 2000 ms, followed by a playing card displayed for 1000 ms. In 72 trials, the card was followed by a probe trial–a card featuring a question mark, prompting the participants to indicate as fast as possible via button press whether the previously presented card had an even or odd value. The task was employed to ensure participants were paying attention to the cards while keeping the manipulation of the cards’ colors as a non-central, task-irrelevant feature. Four of the participants reported recognizing the anomalous nature of the manipulated cards and were excluded from the data analysis because once aware of the manipulation, participants may react differently to subsequent presentations of anomalous stimuli [26].

When calculating reaction times, we excluded incorrect responses and responses that were faster than 200 ms and slower than 2 *SD*s over the individual mean reaction time [52,53].

#### EEG recordings

EEG was recorded using a REFA 72 digital amplifier system (TMSi, Oldenzaal, The Netherlands). EEG signals were recorded with an average reference. We re-referenced the data offline to a calculated average of TP9 and TP10. Using a EEG cap (TMSi), 64 Ag/AgCl-electrodes were placed on following locations (according to international 10–20 system): AF3, AF4, AF7, AF8, AFz, C1, C2, C3, C4, C5, C6, CP1, CP2, CP3, CP4, CP5, CP6, CPz, Cz, F1, F2, F3, F4, F5, F6, F7, F8, FC1, FC2, FC3, FC4, FC5, FC6, FCz, Fp1, Fp2, FT7, FT8, FT9, FT10, Fz, O1, O2, Oz, P1, P2, P3, P4, P5, P6, P7, P8, PO3, PO4, PO7, PO8, POz, Pz, T7, T8, TP7, TP8, TP9 and TP10. Vertical EOG was recorded from sites above and below the left eye. A wet band on the left wrist served as ground electrode and impedances were kept under 50kΩ, which is uncommon yet appropriate for this kind of high-input impedance amplifier [54]. Data collection was controlled by Polybench software 1.25 (TMSi). EEG data were recorded at 512 Hz sampling rate. Offline analysis of the EEG data was conducted using BrainVisionAnalyzer (BrainProducts, Inc.).

For the time-frequency analyses, segments ranging from -2000 to 1000 ms relative to stimulus onset were created. Segments containing eye artifacts were corrected using Gratton & Cole’s method [55], and epochs containing artifacts were rejected automatically using the same settings as mentioned above. Time-frequency decomposition was performed by applying a Morlet complex with 40 linear frequency steps (1–40 Hz) with a Morlet parameter of 5, to trials containing normal and anomalous cards; the resulting wavelets were averaged for each of the conditions. For the analysis of the alpha frequency band, the wavelet layers with 8, 9, 10, 11, and 12 Hz central frequency were extracted and averaged across parieto-occipital and occipital sites (PO3, PO4, PO7, PO8, POz, O1, O2, and Oz). For the baseline alpha power, the three-min signal was segmented in 2000 ms segments (1500 ms overlap) and subjected to the same transformation process as described above.

For the exploratory ERP analyses, the data were band-pass filtered (0.1–30 Hz, 24 dB/oct slope) and resampled to 256 Hz. For the single trials, epochs ranging from -200 to 1000 ms relative to stimulus onset were created, rendering a total of 180 segments per subject. Epochs containing eye blinks were corrected using Gratton & Cole’s method [55]. Epochs with extreme values (changes >100 μV within 100 ms, or values > 100 μV or < -100 μV) were rejected. ERPs were calculated by averaging the signals of normal card trials and reversed card trials, separately. Finally, ERPs were baseline-corrected (-200 ms until stimulus onset). Amplitudes of the early ERP peaks were extracted after performing semiautomatic peak detection: for the P1, local positive maxima were searched 65–100 ms post stimulus onset and pooled (α = .94) over eight posterior sites (POz, PO3, PO7, PO4, PO8, Oz, O1, O2); for the N1, local negative maxima were searched between 110–150 ms post stimulus onset and pooled over four sites (POz, Oz, O1, O2). For the late positive potential (LPP), we scored the mean amplitude 400–800 ms post stimulus onset over the centro-parietal sites P1, P2, Pz, CP1, CP2, and CPz, since LPP is typically most prominent over these sites [6,56]. P300 was analyzed by collapsing the parietal sites P3, Pz, and P4 in the time window 200–400 ms post stimulus onset [57].

To investigate the influence of perceptual anomalies and political attitude strength on ERP amplitudes and prestimulus alpha power, we entered experimental group (anomalous card group vs. normal card group), attitude strength, and their interaction term as predictors into multiple regression models using the *pequod* package for R [58]. A sensitivity analysis using G*Power [59] revealed that the present sample size of 191 participants provided 96% power to detect an effect size of *f*^*2*^ = 0.10 for the moderation effect.

## Results

### Behavioral results

Overall, participants gave mostly correct answers to the probe trials, *M* = 94%, *SD* = 9.7%. There was no difference in accuracy between the experimental groups; *t*(188.8) = -0.62, *p* = .53, Cohen’s *d* = 0.09. Furthermore, the accuracy of participants in the experimental condition did not differ as a function of whether the probe trials featured a normal or an anomalous card; *t*(91) = -1.24, *p* = .22, Cohen’s *d* = 0.13.

The comparison of reaction times in correctly answered probe trials prompting a button press indicated that there was a significant difference between subjects in the experimental and the control group; *t*(187.02) = 1.99, *p* = .048, Cohen’s *d* = 0.29. Participants in the experimental group who were presented with both normal and anomalous cards (*M* = 732 ms, *SD* = 361 ms) reacted slightly faster to the probe trials than participants in the control group (*M* = 835 ms, *SD* = 350 ms). Comparing the reaction times *within* the experimental group, reaction times for normal and anomalous cards did not differ significantly (*M*_*normal*_ = 732 ms, *SD* = 361 ms; *M*_*anomalous*_ = 726 ms, *SD* = 349 ms; *t*(91) = 1.04, *p* = .30, Cohen’s *d* = 0.11). Thus, experimental group and card type did not affect task accuracy but had a small but significant effect on speed.

### EEG results

#### Alpha frequency

We investigated the moderating effect of attitude strength on the relationship between the experimental manipulation and alpha power in the prestimulus interval (2000–0 ms relative to stimulus onset). We entered card condition group as predictor (0 = normal cards only; 1 = normal and anomalous cards), and attitude strength as moderator in a linear regression model; we controlled for the individual differences in resting alpha power by adding baseline alpha power as covariate of no interest (β = 0.89, *t*(158) = 26.64, *p* < .0001). To control for the influence of data collection wave, we also entered the data collection time as a between-subjects factor.

**Fig 4:**
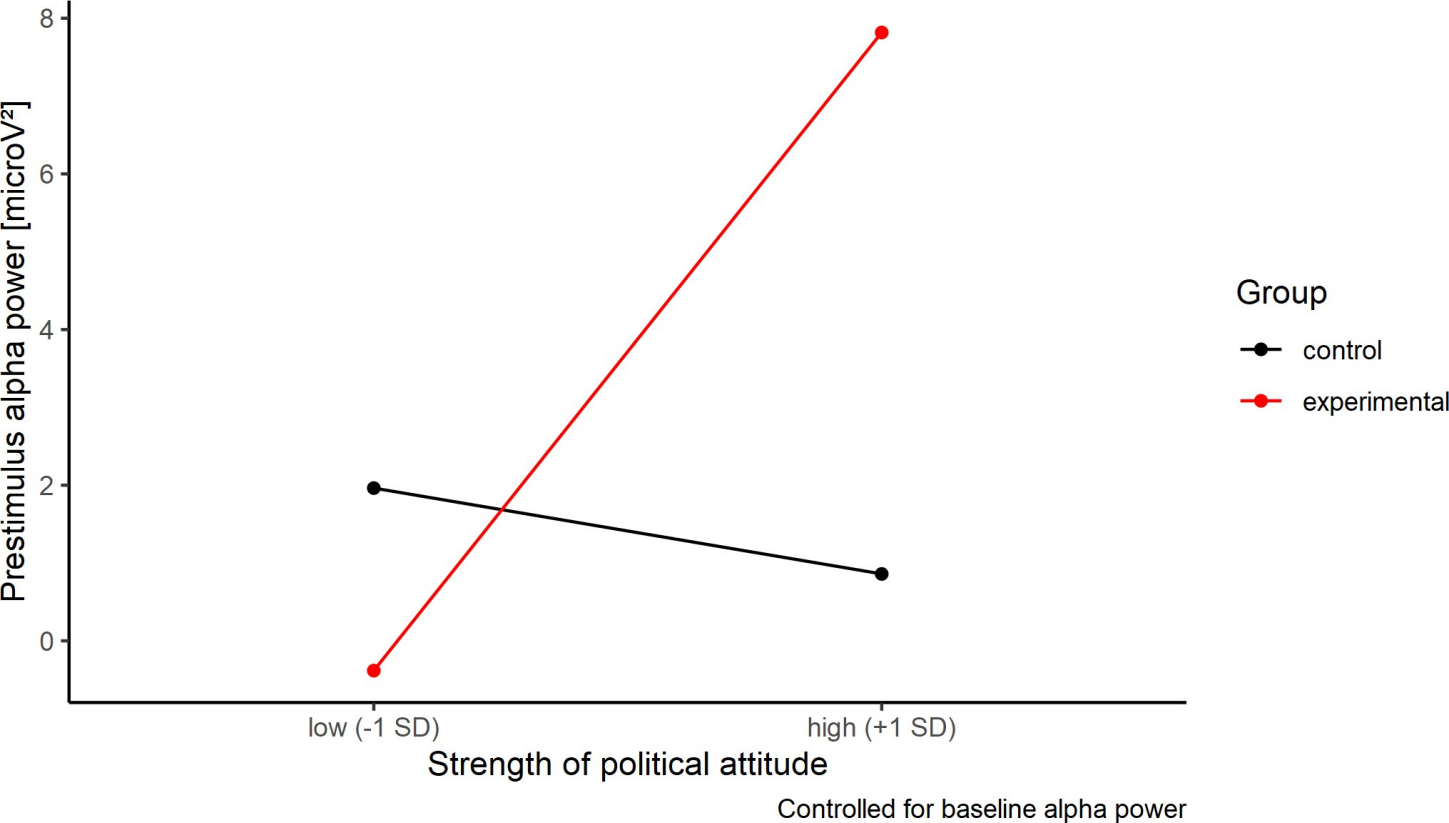
Alpha power simple slopes. Simple slope analysis of pre-stimulus alpha power (8–13 Hz) as a function of ethnocentric attitude strength for participants in the experimental vs. control group, averaged across PO3, PO4, PO7, PO8, POz, O1, O2, and Oz. Baseline alpha power is covaried out.

Participants in the experimental condition showed more pre-stimulus alpha power than those in the control condition, β = 0.10, *t*(158) = 2.10, *p* = .037; attitude strength did not predict alpha power, β = -0.06, *t*(158) = -1.07, *p* = .286. Most importantly, we found a significant condition × attitude strength interaction, β = 0.16, *t*(158) = 2.14, *p* = .034, *f*^*2*^ = 0.07. With the present sample size, we achieved a power of β = .92 to detect the interaction effect (GPower; Faul et al., 2007). A simple slope analysis revealed that the interaction effect was mainly due to alpha power differences between the control and experimental condition in participants high in attitude strength, *b* = 8.20, *t* = 2.97, *p* = .003; in participants with moderate attitudes, there were no differences between the experimental groups, *b* = -1.10, *t* = -0.39, *p* = .70; see Fig 4.

There was a significant interaction between experimental group and data collection time, β = -0.15, *t*(158) = -2.49, *p* = .014. Simple slopes analysis of this two-way interaction revealed that in the first data wave, participants in the experimental group showed higher alpha power than those in the control group; *t*(158) = 2.10, *p* = .037. In the second wave, this was not the case; *t*(158) = -1.38, *p* = .170.

There was no significant three-way interaction effect between the experimental group, attitude strength, and data wave, β = -0.11, *t*(158) = -1.40, *p* = .164, indicating that the time of data collection did not influence the moderating effect of political attitude strength on alpha power.

In a mixed linear model, we analyzed alpha power within participants in the experimental group with attitude strength and baseline alpha power as random effects. Fixed effects showed that there was no effect of card type on prestimulus alpha power, *b* = 0.38, *t*(85.09) = 0.86, *p* = .39, but a significant effect of attitude strength, *b* = 1.42, *t*(9.84) = 2.79, *p* = .020. Card type did not interact with attitude strength, *b* = -0.12, *t*(85.09) = -0.35, *p* = .73. Thus, while there were no differences between normal and anomalous cards, strength of political attitude predicted higher prestimulus alpha power in the experimental condition.

In addition, we performed the same analysis of prestimulus alpha power with absolute ethnocentrism as predictor. The details of the regression model can be found in S1 Supplementary Analyses. In short, there was a marginally significant first-order effect of ethnocentrism on prestimulus alpha power (β = 0.14, *t*(158) = 1.95, *p* = .053), but no other first-order or interaction effects. Thus, while there were differences in alpha power between individuals low and high in ethnocentrism, these differences were not influenced by the presence of anomalies.

#### Exploratory ERP analyses

**Fig 5:**
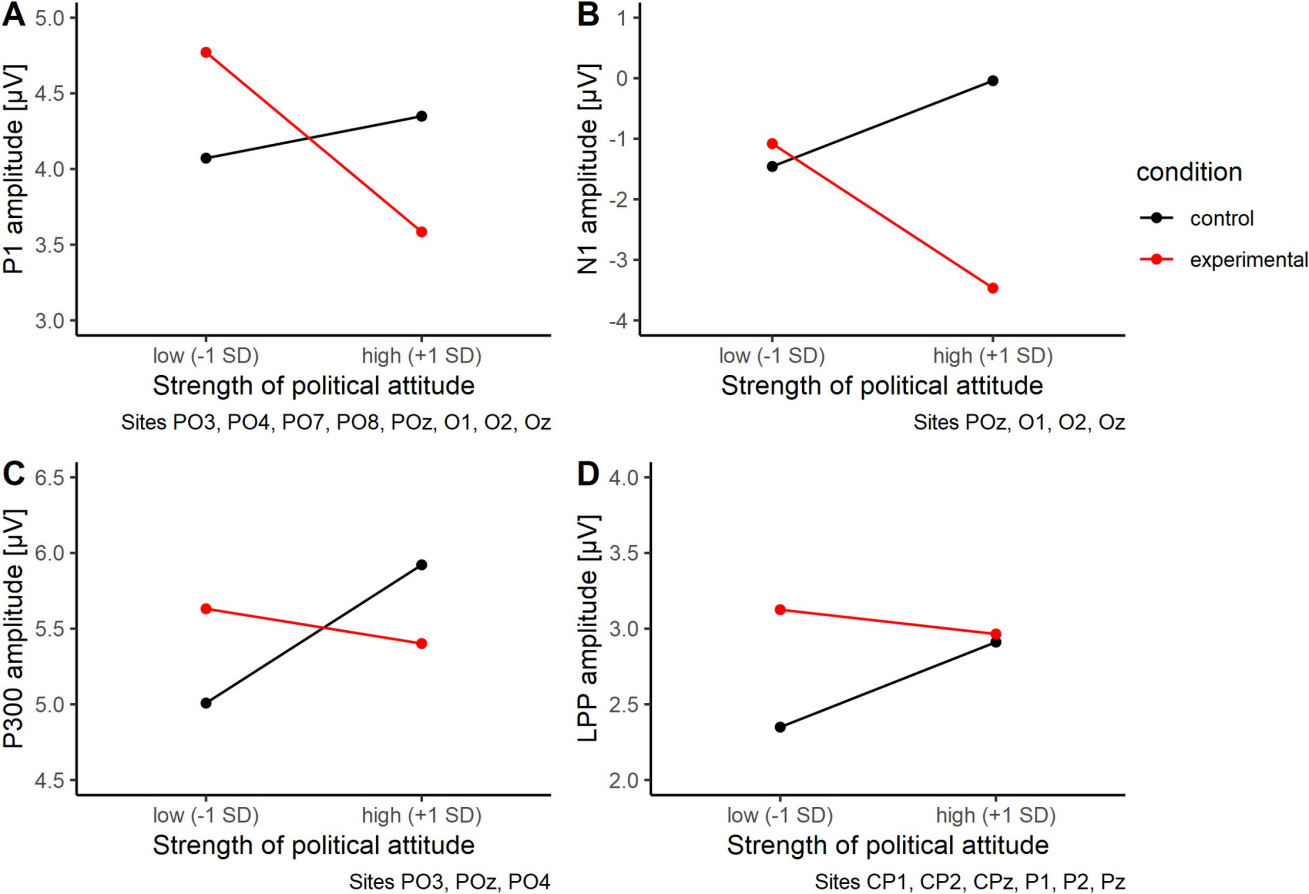
ERP simple slopes. Exploratory simple slope analyses of ERP components. (A) P1, (B) N1, (C) P300, and (D) LPP amplitudes as a function of ethnocentric attitude strength (1 SD below and above sample mean) and card group.

P1. Analysis of the P1 component revealed no significant effects of experimental group (β = -0.01, *t*(159) = -0.10, *p* = .92), attitude strength (β = 0.04, *t*(159) = 0.30, *p* = .76), or data set (β = 0.03, *t*(159) = 0.30, *p* = .76). There was no moderating effect of attitude strength on P1 amplitude, β = -0.16, *t*(159) = -0.87, *p* = .39. There were also no two-way or three-way interactions between the predictors (all β < .12, *t* < 0.86, *p* > .38). Simple slopes of the experimental group * attitude strength interaction can be seen in Fig 5A.

The within-subjects analysis using a linear mixed model showed no effect of card type, attitude strength, nor their interaction on P1 amplitudes in participants in the experimental group (all *b* < .30, *t* < 0.68, *p* > .50).

N1. Analysis of the N1 component revealed a marginal effect of experimental group (β = -0.20, *t*(157) = -1.75, *p* = .083). Participants in the experimental group showed marginally stronger N1 amplitudes than those in the control group. There was no first-order effect of attitude strength (β = 0.17, *t*(157) = 1.19, *p* = .23), but a significant effect of data set (β = -0.26, *t*(157) = -2.35, *p* = .020), indicating that in the second data wave, participants showed higher N1 amplitudes than in the first one. There was a marginally significant interaction between attitude strength and experimental group, β = -0.30, *t*(157) = -1.68, *p* = .096 (see Fig 5B). Participants with stronger (vs. moderate) attitudes showed increased N1 amplitudes in the experimental group (vs. control group).

The within-subjects analysis using a linear mixed model showed no effect of card type, attitude strength, nor their interaction on participants’ N1 amplitudes in the experimental group (all *b* < .64, *t* < 1.09, *p* > .28).

P300. Amplitude of the P300 component was not influenced by experimental group, attitude strength, or data set. Furthermore, two-way and three-way interactions were not significant, all β < .15, *t* < 1.10, *p* > .27. Importantly, there was no moderating effect of attitude strength on P300 amplitude, β = -0.12, *t*(161) = -0.65, *p* = .52 (Fig 5C). There were also no differences in P300 amplitude when comparing normal and anomalous cards within the experimental group, all *b* < 0.15, *t* < 0.89, p > .37.

**Fig 6:**
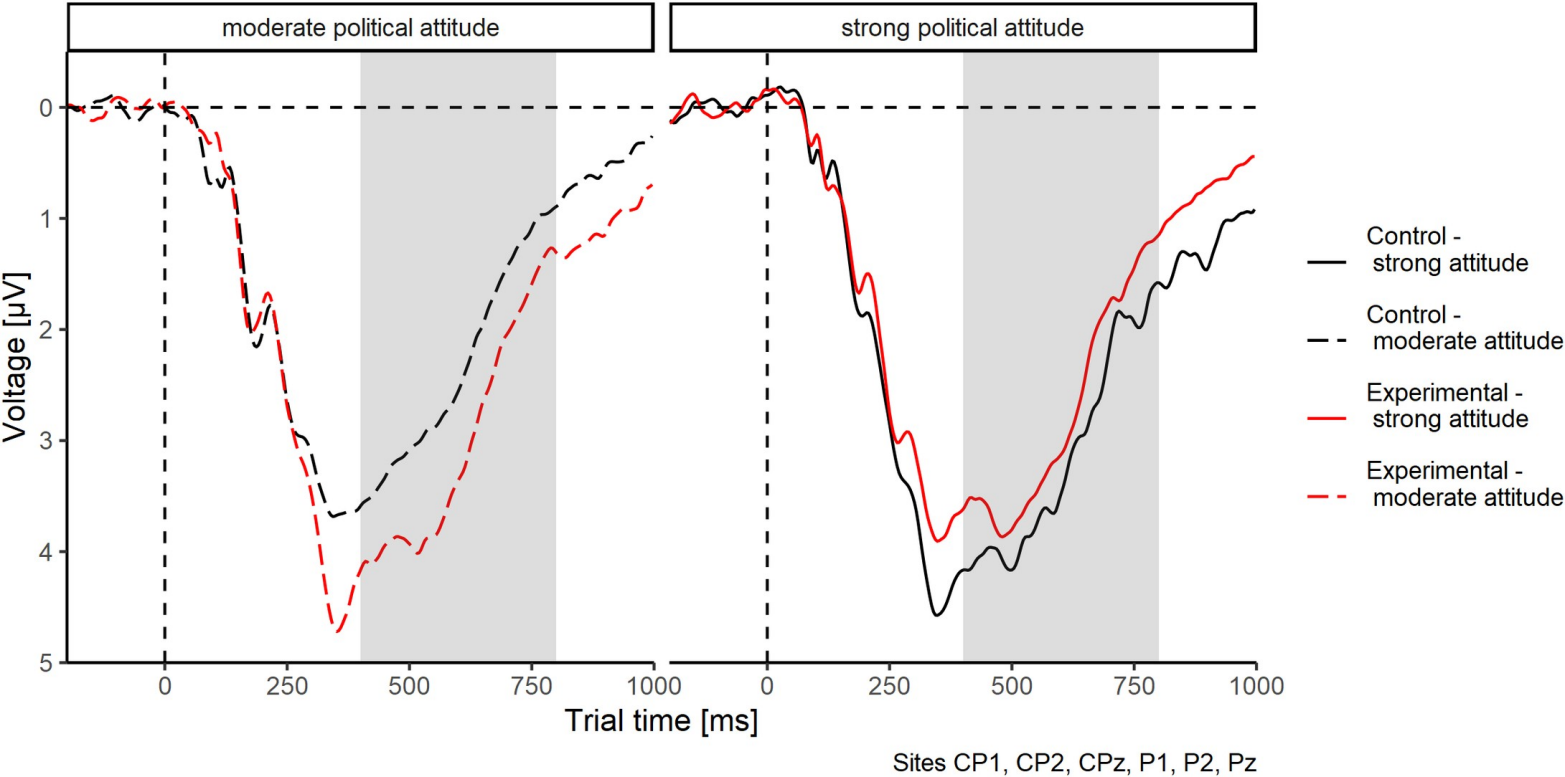
Moderated ERP curve. Calculated for participants high (+1 SD) and low (-1 SD) in ethnocentric attitude strength, separate for participants in the experimental group (normal and anomalous cards) and control group (normal cards only). Values are averaged across centroparietal and parietal electrodes, as used for LPP analysis. LPP time window (400–800 ms post stimulus onset) is depicted by the gray rectangle.

Late positive potential (LPP). LPP amplitude was not predicted by attitude strength; β = 0.16, *t*(160) = 1.08, *p* = .28. There was no significant influence of experimental group on LPP amplitude; β = 0.12, *t*(160) = 0.95, *p* = .32. The interaction, that is, the moderating effect of attitude strength, was also not significant; β = -0.13, *t* = -0.71, *p* = .48, *f*^*2*^ = .01; see Fig 5D. Against our expectations, participants with moderate ethnocentric attitudes did not react to perceptual anomalies with more event-related centroparietal cortical excitation (for a moderated ERP curve, see Fig 6). There were also no effects of data set or its interactions (all *t*(160) < 0.60, *p* > .55). The analysis of LPP amplitude within the experimental group using linear mixed modelling revealed no effects of card type (normal vs. anomalous), attitude strength, or their interaction (all *b* < .11, *t* < 1.17, *p* > .24).

All ERP components were also analyzed with absolute ethnocentrism as predictor instead of attitude strength. The results are available in S1 Supplementary Analyses.

## Discussion

This research was conducted to test the hypothesis that individuals with strong political attitudes react to expectancy-violating stimuli by suppressing them. We argue that this suppression manifests as a tonic increase in alpha oscillations in the brain. Indeed, we found that strength of political belief moderated prestimulus alpha power in response to perceptual anomalies: in participants with strong political attitudes, alpha power was higher in the presence of reverse-colored playing cards than in the control condition; this difference was absent in individuals with moderate beliefs.

This intriguing finding is in congruence with the argument of Inzlicht et al. [9] that ideologically committed individuals gate their attention away from potential sources of uncertainty. The size of the effect is comparatively small. However, since the manipulation is de facto subliminal (or at least periliminal), the small effect sizes are not surprising. In fact, Randles and colleagues [60] investigated the effect sizes of classic expectancy violation paradigms on behavioral outcomes (here, working memory performance) with Bayesian inferences, revealing small but meaningful effect sizes.

Based on evidence that politically extreme participants exhibited smaller pupil dilation responses to reverse-colored playing cards than moderate participants [25], we investigated whether the strength of political attitudes is also related to reduced phasic neurophysiological responses (i.e., event-related potentials) to reverse-colored playing cards. This prediction was not supported, as we did not find a parallel effect of political attitude strength on the amplitudes of the P1, P300, and the late positive potential (LPP). One reason may be that stimulus-evoked EEG responses (i.e., ERPs) and stimulus-evoked pupil dilation responses are not consistently correlated [41,61]. The two phenomena seem to be related in a complex way, with the largest evoked EEG responses emerging at intermediate levels of pupil diameter, not evoked pupil dilation [40,61]. Thus, it is conceivable that reverse-colored playing cards evoke phasic pupil dilation responses in the absence of phasic electrophysiological responses. We did find a marginal moderation effect of attitude strength and experimental group on N1 amplitude. This result is unsurprising given that the P1/N1 complex is at least partially generated by alpha power [62,63], and N1 amplitude often correlates with prestimulus alpha power, albeit in a visual paradigm [64].

Previous research suggests that conservatives show greater reactions to arousing stimuli [6] or generally have a lower threshold for emotional arousal [65]. However, in the present paper our focus was on the strength of attitudes, that is, the deviation from the group mean in an ethnocentrism questionnaire, regardless of political orientation. Our sample consisted of mostly liberal participants, that is, university students (cf. [25]): the ethnocentrism scores in our sample were well below the scale mean of 3.5 and ranged from 1 to 4.40 (*M* = 2.08). Sleegers and colleagues reported that both extremely liberal and conservative participants reacted with intolerance when their worldviews were challenged [13,26]. Indeed, it seems attentional processes varied depending on the strength of ideological convictions [66]. We can only speculate how a more evenly distributed sample would have influenced the results. However, the finding that the results are present even in our quite liberal sample further validates the strategy of studying extremism as a deviation from the norm.

An explanation for why we found tonic but not phasic neurophysiological reactivity to perceptual anomalies may be the subtleness and task-irrelevance of our manipulation (i.e., presenting reverse-colored playing cards). Conscious and task-relevant conflict, threats, discrepancies, and meaning violations typically evoke phasic reactivity, a frequent epicenter of which is the dorsal anterior cingulate cortex (dACC) [16,21]. Dehaene and colleagues found that only conscious detection of inconsistencies evokes ACC activation [67,68]. Although we cannot equate ACC activation with evoked EEG responses [57,69], the implicit nature of our manipulation may nevertheless have prevented other phasic electrocortical responses.

### Limitations and future research

This experiment adds electrophysiological evidence to the behavioral and pupillometric findings from past research. We have shown that ideologically convicted, but not moderate participants, show tonic increases in prestimulus alpha power in reaction to anomalous playing cards. Future research should expand on this finding with different manipulations.

One potential factor limiting the generalizability of our findings is the measure of political attitude employed in this research: given the sociopolitical circumstances during the conceptualization of the study, we used a measure of ethnocentrism as a proxy of socio-political attitude. To increase generalizability of our findings, we suggest future research include both general measures of political attitudes and attitudes in other political domains.

Our research is correlative, we thus cannot infer causal relationships between attitude strength and electrophysiological reactions. There are two possible lines of causality that could explain the obtained results. Either individuals with stronger attitudes show increased attentional gating of possible anomalies, or individuals with decreased reactivity to expectancy violations hold extreme beliefs more easily. In this view, those who notice and react strongly to anomalies are more likely to update their belief systems, possibly leading to more moderate attitudes. This *chicken and egg* problem, however, does not take away from our findings. Future research should investigate the direction of causality in the development of political conviction.

In our study, we did not assess participants’ prior experience with playing cards (e.g., playing poker, casino visits) as a potential moderator of the observed effects. However, the fact that the anomaly was only detected by four participants and the absence of behavioral effects lead us to believe that experience with playing cards had no influence on the results. Future studies should, however, investigate this assumption empirically.

Given that conscious and unconscious meaning violations seem to produce different neural effects, future studies may also contrast them directly. Participants usually do not detect the anomalous playing cards, which makes the manipulation especially suitable for creating unconscious expectancy violations. Informing participants about the presence of reverse-colored playing cards would turn these otherwise undetected perceptual anomalies into conscious ones, thus allowing also for a conscious manipulation of meaning violations. The danger associated with making them conscious is that participants may quickly accommodate their existing meaning frameworks about playing cards in such a way that hearts can also be black and spades can also be red, which would remove the expectancy-violating aspect from these previously anomalous cards. A possible solution could be to use logically impossible meaning violations (e.g., Bill is both married and a bachelor).

For our research question, a between-subjects design was a more reasonable choice than a within-subjects design: A within-subjects design where participants complete both the experimental and the control condition may render higher statistical power but carries an important caveat: detection of the anomaly may influence subsequent processing of the anomalous stimuli [25,70]. Indeed, the within-subjects analyses in the present paper show that the presence of anomalous stimuli in the task had effects on both the anomalous and the regular control stimuli [25,63].

### Conclusion

We found that the processing of perceptual anomalies is influenced by the strength of an individual’s convictions, and that this influence is probably due to tonic attentional suppression. Notably, this effect was linked to the strength of ethnocentric attitudes. Thus, convinced liberals may gate attention away from expectancy-violating information just as readily as convinced conservatives.

## Supporting information

S1 Supplementary Analyses Moderating effects of ethnocentrism on EEG measures in reaction to perceptual anomalies. (DOCX)

S1 Dataset Dataset and R script underlying the analyses in the manuscript. (ZIP)
