# Supplementary material for: Strength of socio-political attitudes moderates electrophysiological responses to perceptual anomalies
Source: PLoS One. 2019 Aug 5;14(8):e0220732. doi: 10.1371/journal.pone.0220732 (PMC6681971; doi:10.1371/journal.pone.0220732)
Supplement: S1 Supplementary Analyses — (DOCX) [file pone.0220732.s001.docx]

**S1 Supplementary Analyses.** Moderating effects of ethnocentrism on EEG measures in reaction to perceptual anomalies.

**Alpha Frequency.** In addition to the effect of attitude strength, that is the deviation from the sample mean, we analyzed the relationship between absolute ethnocentrism values and card condition group. As in the previous analysis, we included baseline alpha power as covariate to account for individual differences (β = 0.91, *t*(158) = 27.50, *p* < .0001). We also entered data collection time as a between-subjects factor to control for the effect of time.


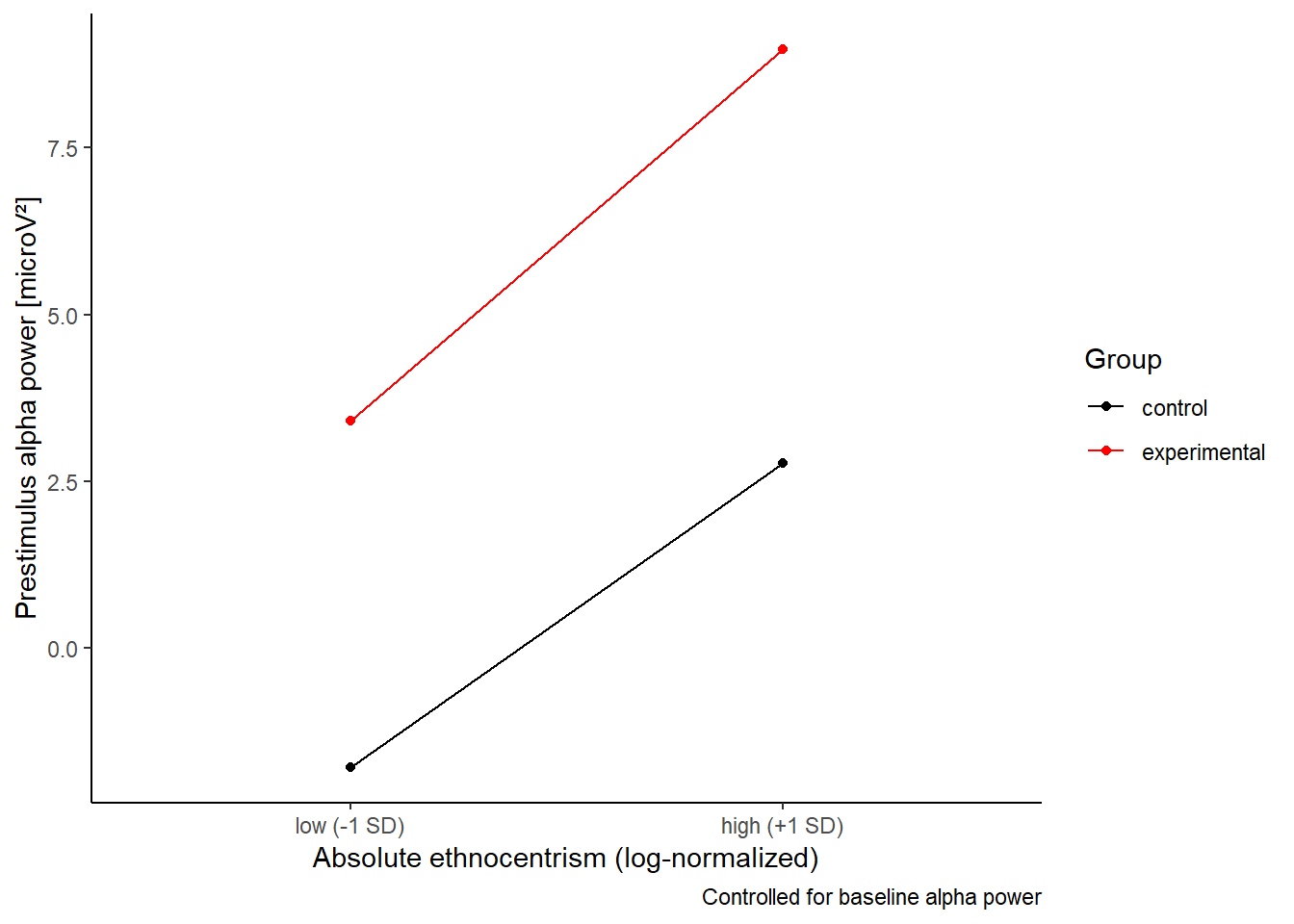
 There was no first-order effect of card condition on prestimulus alpha power, β = 0.11, *t*(158) = 0.96, *p* = .34. The effect of ethnocentrism on alpha power was marginally significant, β = 0.14, t(158) = 1.95, p = .053. Most importantly, there was no interaction effect between card condition and ethnocentrism, β = 0.03, *t*(158) = 0.26, *p* = .79. This indicates that while there was a marginally significant difference in alpha power between high and low ethnocentric individuals, our experimental condition did not affect this difference. Furthermore, there was a significant first-order effect of data collection time, indicating that in the second wave of data collection, alpha power was higher than in the first one, β = 0.24, *t*(158) = 2.12, *p* = .036. No other first-, second-, or third-order interaction effects approached significance (β < .17, *p* > .19).

|  | **Alpha Power** | | | |
| --- | --- | --- | --- | --- |
| *Predictors* | *b* | β | *t* | *p* |
| (Intercept) | -4.81 |  | -1.50 | 0.135 |
| Alpha Base | 0.83 | 0.91 | 27.50 | **<0.001** |
| Cards | 3.97 | 0.11 | 0.96 | 0.341 |
| Ethnocentrism | 8.42 | 0.14 | 1.95 | 0.053 |
| Data | 9.01 | 0.24 | 2.12 | **0.036** |
| Cards * Ethnocentrism | 1.63 | 0.03 | 0.26 | 0.793 |
| Cards * Data | -2.95 | -0.07 | -0.51 | 0.608 |
| Ethnocentrism * Data | -7.42 | -0.17 | -1.31 | 0.193 |
| Cards * Ethnocentrism * Data | -5.99 | -0.12 | -0.75 | 0.455 |
| Observations | 167 | | | |
| R^2^ / R^2^ adjusted | 0.842 / 0.834 | | | |

**ERP analyses**

**P1.** P1 amplitudes were not significantly influenced by ethnocentrism, card condition, data collection time, or any two- or three-way interactions; all β < .23, *t* < 1.33, *p* > .18.


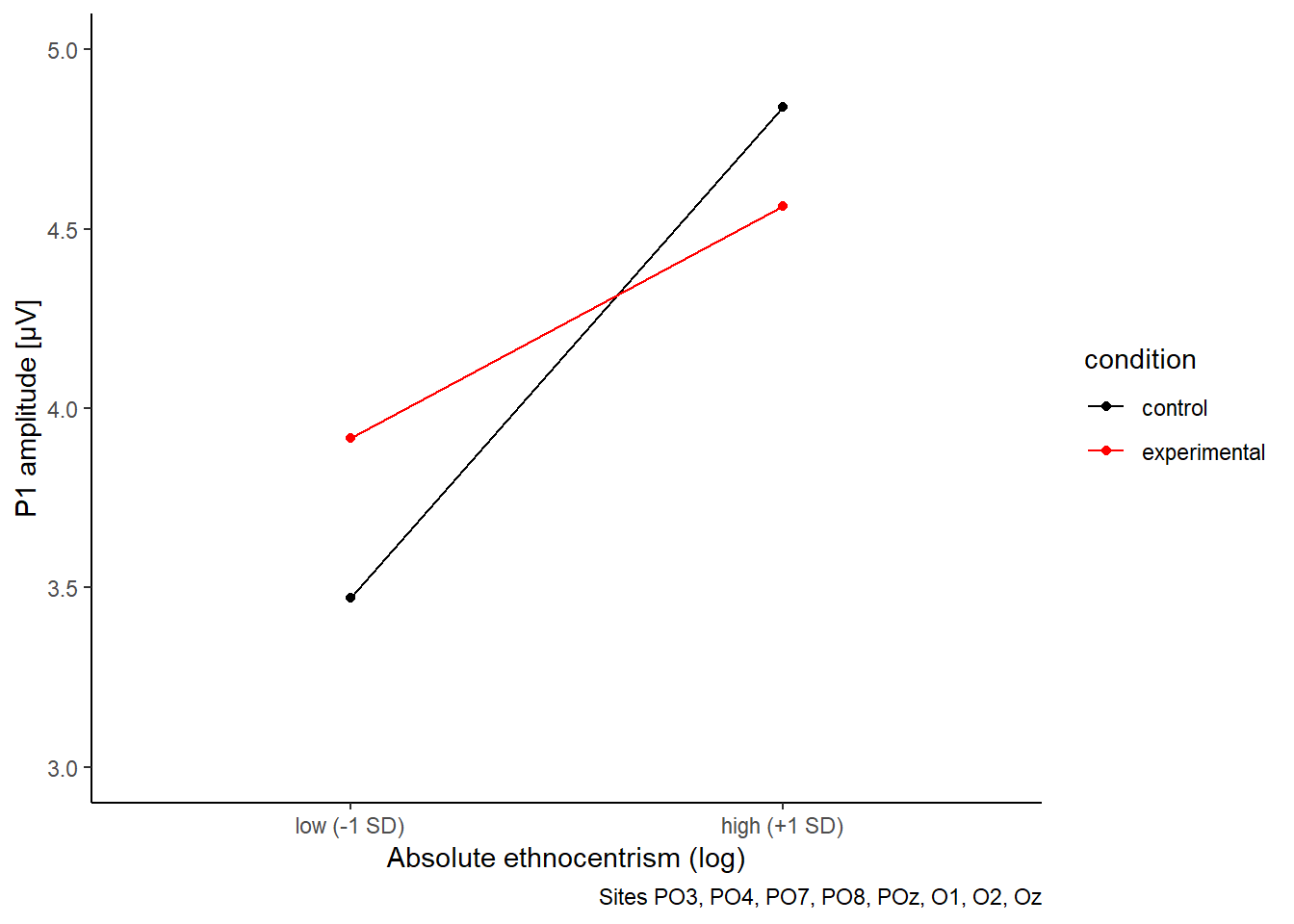


|  | **P1 amplitude** | | | |
| --- | --- | --- | --- | --- |
| *Predictors* | *b* | β | *t* | *p* |
| (Intercept) | 2.68 |  | 2.20 | **0.030** |
| Cards | 0.87 | 0.15 | 0.53 | 0.593 |
| Ethnocentrism | 2.20 | 0.23 | 1.33 | 0.187 |
| Data | 0.78 | 0.13 | 0.47 | 0.638 |
| Cards * Ethnocentrism | -1.16 | -0.16 | -0.48 | 0.630 |
| Cards * Data | -0.30 | -0.05 | -0.13 | 0.895 |
| Ethnocentrism * Data | -0.89 | -0.13 | -0.40 | 0.689 |
| Cards * Ethnocentrism * Data | -0.89 | -0.11 | -0.28 | 0.778 |
| Observations | 164 | | | |
| R^2^ / R^2^ adjusted | 0.034 / -0.010 | | | |

**N1.** The analysis of socio-political attitudes as predictors of N1 amplitudes to anomalous playing cards revealed no significant effects of card group, ethnocentrism, data collection time, or any interactions; all β < .50, *t* < 1.53, *p* > .13.


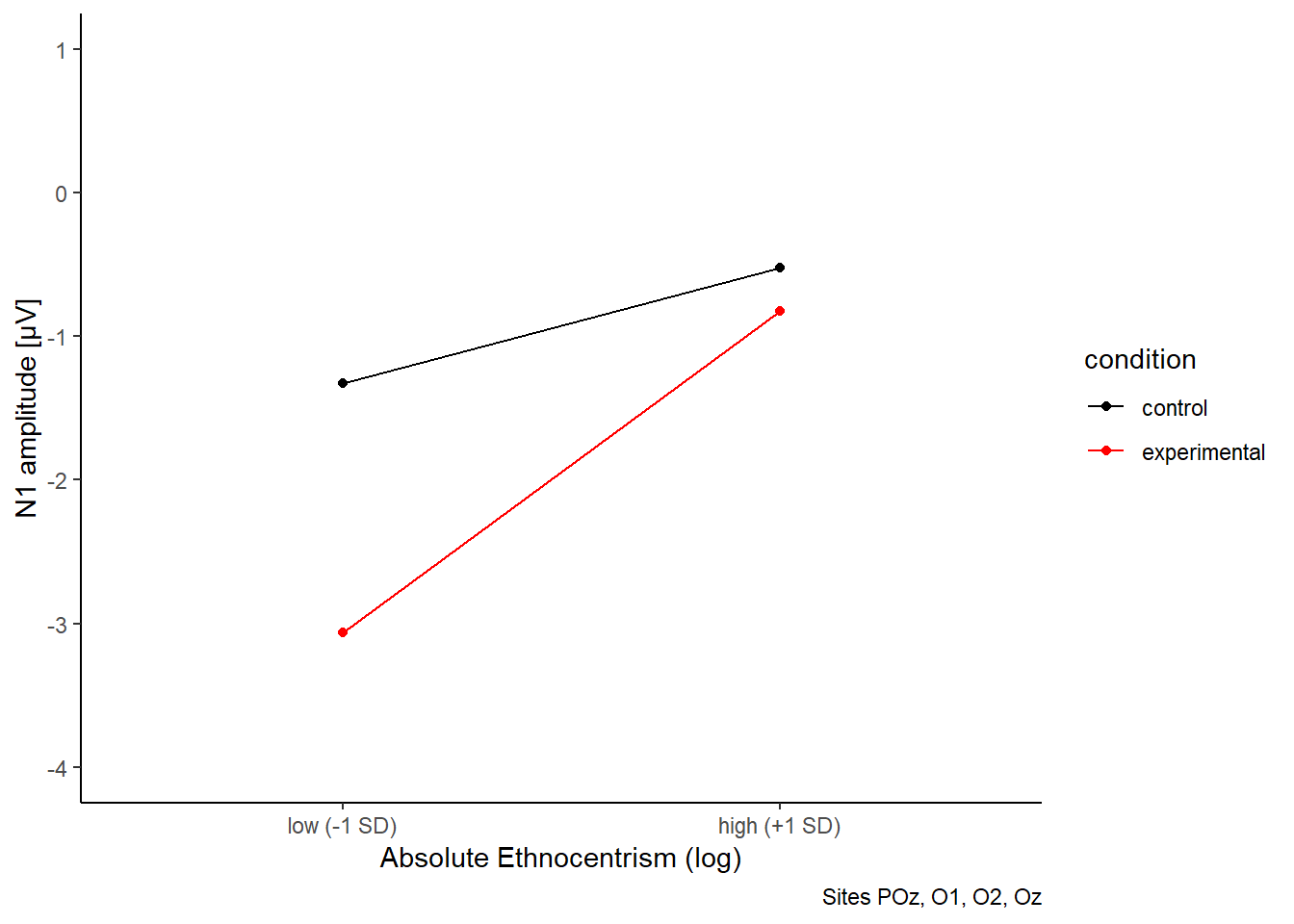


|  | **N 1 amplitude** | | | |
| --- | --- | --- | --- | --- |
| *Predictors* | *b* | β | *t* | *p* |
| (Intercept) | -1.84 |  | -1.11 | 0.267 |
| Cards | -2.58 | -0.32 | -1.18 | 0.240 |
| Ethnocentrism | 1.34 | 0.10 | 0.60 | 0.552 |
| Data | -2.29 | -0.29 | -1.02 | 0.309 |
| Cards * Ethnocentrism | 2.33 | 0.23 | 0.71 | 0.476 |
| Cards * Data | 4.68 | 0.52 | 1.53 | 0.129 |
| Ethnocentrism * Data | 0.54 | 0.06 | 0.18 | 0.857 |
| Cards * Ethnocentrism * Data | -4.72 | -0.43 | -1.11 | 0.268 |
| Observations | 164 | | | |
| R^2^ / R^2^ adjusted | 0.056 / 0.013 | | | |

**P300.** The analysis of the P300 amplitude over parieto-occipital sites revealed no significant influence of card condition (β = -0.01, *t*(158) = -0.06, *p* = .96), ethnocentrism (β = 0.14, *t*(158) = 0.84, *p* = .40), data collection time (β = 0.16, *t*(158) = 0.56, *p* = .58), or their interactions (all β < .61, *t* < 1.58, *p* > .12).


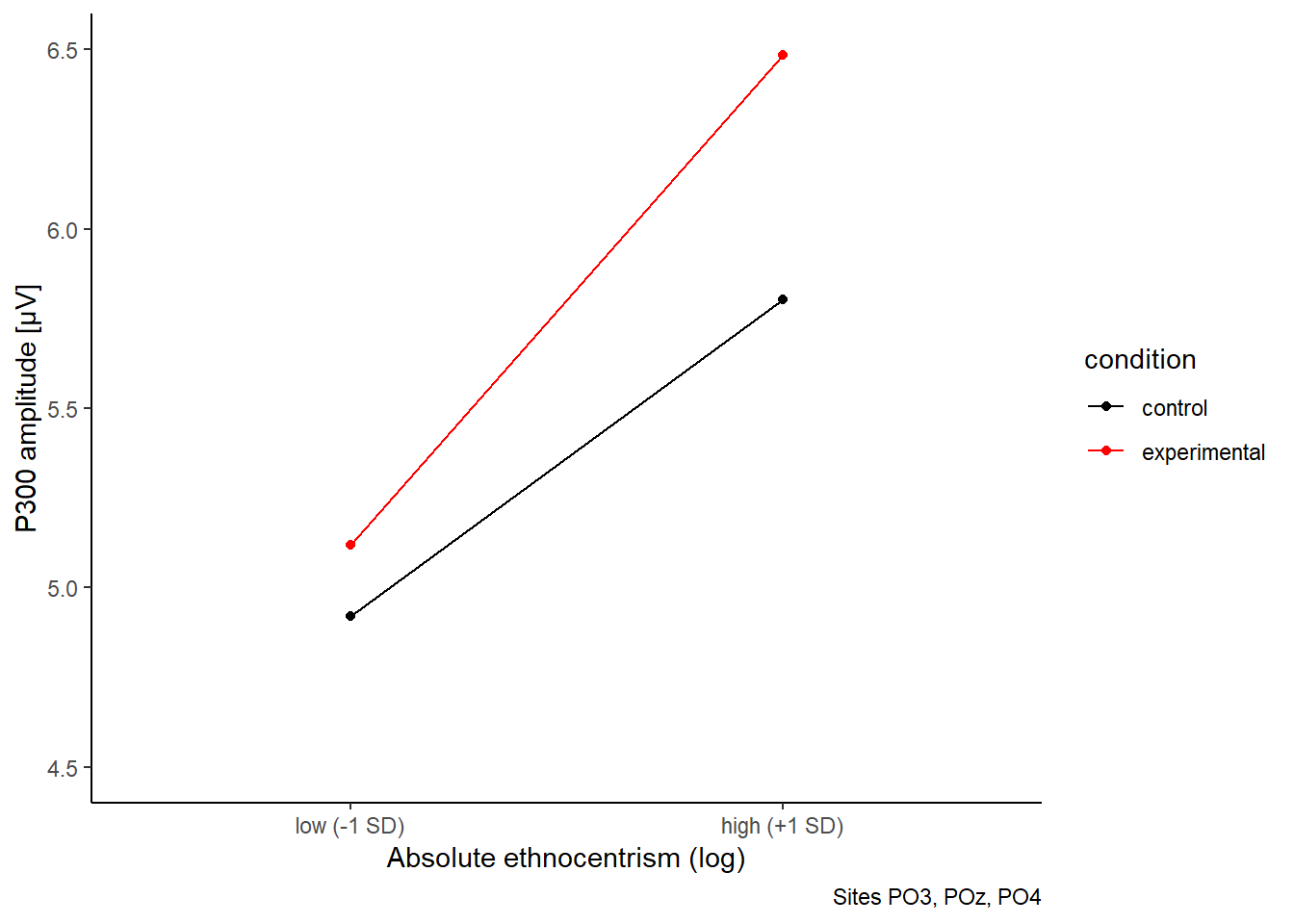


|  | **P3 amplitude** | | | |
| --- | --- | --- | --- | --- |
| *Predictors* | *b* | β | *t* | *p* |
| (Intercept) | 4.40 |  | 3.48 | **0.001** |
| Cards | -0.08 | -0.01 | -0.05 | 0.960 |
| Ethnocentrism | 1.44 | 0.14 | 0.84 | 0.403 |
| Data | 0.96 | 0.16 | 0.56 | 0.575 |
| Cards * Ethnocentrism | 0.78 | 0.10 | 0.31 | 0.755 |
| Cards * Data | 2.77 | 0.41 | 1.19 | 0.236 |
| Ethnocentrism * Data | -0.23 | -0.03 | -0.10 | 0.920 |
| Cards * Ethnocentrism * Data | -5.10 | -0.62 | -1.58 | 0.117 |
| Observations | 166 | | | |
| R^2^ / R^2^ adjusted | 0.057 / 0.015 | | | |

**Late positive potential**. The analysis of the influence of absolute ethnocentric attitudes on the late positive potential (LPP) revealed no effect of card group, β = 0.27, *t*(158) = 1.00, *p* = .32. There was no significant effect of ethnocentrism (β = 0.01, *t*(158) = 0.03, *p* = .97), and no interaction effect of card group and ethnocentrism (β = -0.20, *t*(158) = -0.64, *p* = .52). There was an effect of time of data collection, and a marginal interaction effect card group * ethnocentrism * data collection. The results do not suggest that the direction of socio-political attitude had an effect on LPP amplitudes in reaction to anomalies.


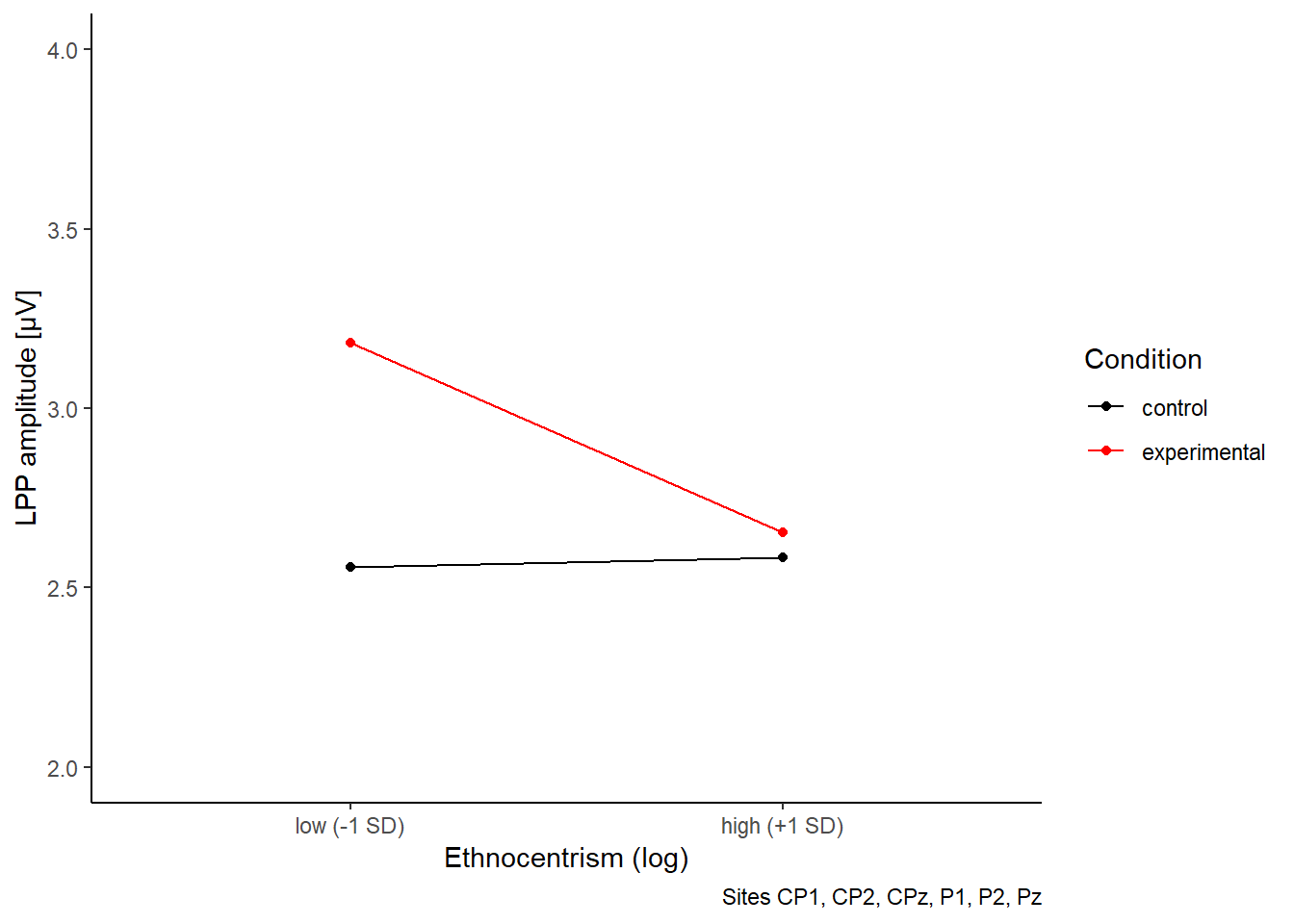


|  | **LPP amplitude** | | | |
| --- | --- | --- | --- | --- |
| *Predictors* | *b* | β | *t* | *p* |
| (Intercept) | 2.55 |  | 3.58 | **<0.001** |
| Cards | 0.95 | 0.27 | 1.00 | 0.316 |
| Ethnocentrism | 0.03 | 0.01 | 0.03 | 0.974 |
| Data | 2.42 | 0.68 | 2.49 | **0.014** |
| Cards * Ethnocentrism | -0.90 | -0.20 | -0.64 | 0.525 |
| Cards * Data | -2.34 | -0.59 | -1.77 | 0.079 |
| Ethnocentrism * Data | -3.18 | -0.77 | -2.47 | **0.015** |
| Cards * Ethnocentrism * Data | 3.08 | 0.64 | 1.68 | 0.095 |
| Observations | 165 | | | |
| R^2^ / R^2^ adjusted | 0.100 / 0.060 | | | |
